# Malaria in central Vietnam: analysis of risk factors by multivariate analysis and classification tree models

**DOI:** 10.1186/1475-2875-7-28

**Published:** 2008-01-30

**Authors:** Ngo Duc Thang, Annette Erhart, Niko Speybroeck, Le Xuan Hung, Le Khanh Thuan, Cong Trinh Hung, Pham Van Ky, Marc Coosemans, Umberto D'Alessandro

**Affiliations:** 1 National Institute of Malariology, Parasitology and Entomology, Luong The Vinh street, BC 10200 Tu Liem district, Hanoi, Vietnam; 2 Prince Leopold Institute of Tropical Medicine, Nationalestraat 155, 2000 Antwerp, Belgium; 3 Ecole de santé publique, Université Catholique de Louvain, Clos Chapelle-aux-Champs, 1200 Bruxelles, Belgium; 4 Center of Malariology, Parasitology and Entomology, Ninh Thuan province, 156 Ngo Gia Tu, Phan Rang, Ninh Thuan, Vietnam

## Abstract

**Background:**

In Central Vietnam, forest malaria remains difficult to control due to the complex interactions between human, vector and environmental factors.

**Methods:**

Prior to a community-based intervention to assess the efficacy of long-lasting insecticidal hammocks, a complete census (18,646 individuals) and a baseline cross-sectional survey for determining malaria prevalence and related risk factors were carried out. Multivariate analysis using survey logistic regression was combined to a classification tree model (CART) to better define the relative importance and inter-relations between the different risk factors.

**Results:**

The study population was mostly from the Ra-glai ethnic group (88%), with both low education and socio-economic status and engaged mainly in forest activities (58%). The multivariate analysis confirmed forest activity, bed net use, ethnicity, age and education as risk factors for malaria infections, but could not handle multiple interactions. The CART analysis showed that the most important risk factor for malaria was the wealth category, the wealthiest group being much less infected (8.9%) than the lower and medium wealth category (16.6%). In the former, forest activity and bed net use were the most determinant risk factors for malaria, while in the lower and medium wealth category, insecticide treated nets were most important, although the latter were less protective among Ra-glai people.

**Conclusion:**

The combination of CART and multivariate analysis constitute a novel analytical approach, providing an accurate and dynamic picture of the main risk factors for malaria infection. Results show that the control of forest malaria remains an extremely complex task that has to address poverty-related risk factors such as education, ethnicity and housing conditions.

## Background

Since the launching of its national programme in 1991, Vietnam has been able to successfully control malaria [1]. In 2003, the number of recorded malaria cases was 164,706, an 88% spectacular decrease compared to the 1992 figures. Similarly, malaria deaths have become rare and no epidemic has been recently detected [2]. Such extremely good results were possible by the national scaling up of the use of Insecticide-Treated Nets (ITN) coupled with an important media campaign on the importance of malaria and of using ITN for its prevention. Indoor-residual spraying (IRS) was used only in epidemic prone-areas or where ITN coverage was very low [2]. The recent introduction of Long-Lasting Insecticide Nets (LLINs), although not officially adopted by Vietnam yet, has overcome the problems of low re-treatment rates, washing and variation in insecticide dosing, possibly improving their effectiveness [3,4]. Against this extremely positive background, malaria remains a problem in some geographically limited areas, usually rural and remote, forested and hilly, representing a risk not only for the local population but also for migrant workers from non-endemic areas. Besides the burden on the local population, the risk of spreading malaria from these areas to others, where transmission has virtually stopped, exists. Therefore, though geographically limited, the control of malaria in these areas is extremely important for the whole Vietnam and possibly for its neighbouring countries. Currently, about half of all malaria cases, more than 80% of severe cases and over 90% of malaria-related deaths occur in the central highlands [5-7]. In these areas, the main vector is *Anopheles dirus sensu stricto *(s.s.), a highly anthropophylic sylvatic species, whose exophagy and exophily as well as early biting habits challenge the impact of interventions such as IRS or ITN [8,9]. Indeed, in recent studies carried out in the forested areas of Central Vietnam, forest activity has been identified has a strong risk factor for malaria infection [10,11]. This calls for a new approach targeting forest workers and addressing the behavioural characteristics of the local vector. As hammocks are commonly used in this area, particularly in the forest, the introduction of Long-Lasting Insecticidal Hammocks (LLIH) (locally made hammocks covered with long-lasting insecticidal netting) might be an effective intervention to protect against malaria infections.

A community-based, cluster-randomized trial has been carried out in Ninh Thuan Province in the Centre-South Vietnam, to assess the effectiveness of LLIH in controlling forest malaria. Prior to the intervention, a complete census of the population in the study area as well as a malariometric cross-sectional survey were carried out. This paper presents the socio-demographic and malariometric data at baseline together with a malaria risk factor analysis combining two different, but complementary approaches: multivariate analysis and classification and regression tree (CART).

## Material and methods

### Study site

Ninh Thuan, in the southern end of Central Vietnam, has 568,535 inhabitants (2004 data) and is divided in six districts (Phan Rang – Thap Cham city, Bac Ai, Ninh Son, Ninh Phuoc, Ninh Hai, Thuan Bac). The Ra-glai, Cham, and Kinh are the most common ethnic groups. The study was carried out in two districts, Bac Ai with eight communes and 25 villages and Ninh Son with two communes and five villages, settled mainly by Ra-glai people practising subsistence agriculture (maize, cashew, rice, beans and manioc) and cultivating cash crops such as coffee and cotton [12]. They also exploit forest products (bamboos, resin, and hunting).

The climate is a combination of tropical monsoon and dry and windy weather. The dry season is from January to April, with the coldest period in January and February and the rainy season from May to December. The mean rainfall is 725 mm/year, with the mean temperature ranging between 25°C and 30°C and the humidity between 70% and 80%. Malaria transmission is perennial with two peaks, one in June and the other in October. Twenty two different *Anopheles *species have been identified: the two main vectors are *An. dirus s.s*. (former species A) and *An. minimus A*; secondary vectors, such as *Anopheles maculates, An. jeyporiensis *or *An. pampanai*, may play a non-negligible role in the local malaria transmission.

### Census and cross-sectional survey

A full census of the study population was done in March 2004. Information on age, sex, socio-economic status, forest activity, bed net availability, and previous vector control measures was collected. The census file was routinely updated as births, deaths and migrations were collected by hamlet health workers and reported monthly to the malaria provincial station where the electronic census file was managed. The study area was divided into 20 clusters (about 1,000 inhabitants each) later divided in 10 intervention and 10 control clusters (December 2004). A random sample of 160 individuals aged 10–60 years was selected from each cluster for the baseline survey carried out in April 2004, before the start of the rainy season. The sample size was computed on the basis of the expected effect of LLIH and taking into account the cluster design, i.e. 30% reduction (5% level and 80% power) of malaria sero-prevalence measured by subsequent surveys in this cohort, assuming an initial sero-prevalence of 8–10%.

A questionnaire on malaria symptoms and treatments was administered and clinical examination including collection of body temperature and spleen size was carried out. A blood sample for thick and thin blood film and for later determination of antimalarial antibodies titres was collected on Whatman N°3 filter paper. Suspected malaria cases were treated presumptively with chloroquine or artesunate. A forest worker was defined as a person whose main income was based on forest activities (farmers, hunters, etc...). However, other people had forest activities (hunting, collecting forest products, etc.) without necessarily being a forest worker. Forest activities were quantified by asking the number of days/nights spent in the forest during the month prior to the census. Three categories of forest activities were defined as "no"; "occasional" if people work and sleep sometimes in the forest or "regular work & sleep" when they daily worked and slept in the forest.

### Laboratory tests

Blood slides were stained with a 3% Giemsa solution for 45 minutes. The number of asexual parasites per 200 white blood cells (WBCs) was counted and parasite densities were computed assuming a mean WBC count of 8,000/μL. A slide was defined as negative if no asexual forms were found after counting 1,000 WBCs. Slides were read first at the Provincial Malaria Station and a quality control was carried out at the National Institute of Malariology, Parasitology and Entomology (NIMPE) in Hanoi. Discrepant results were re-read and confirmed by a third technician at NIMPE, Hanoi.

Patients with malaria symptoms prior to microscopic diagnosis were classified as suspected malaria cases. A malaria infection was defined as a positive blood slide with *Plasmodium *asexual forms, regardless of symptoms and parasite density. Clinical malaria was defined as a patient with fever (body temperature ≥ 37.5°C), and/or history of fever in the past 48 hours, and a positive blood slide for *Plasmodium *asexual forms. Malaria prevalence was computed regardless of the species differentiation.

### Data management and statistical analysis

Data were double entered, checked and cleaned using EpiInfo v6.04d. The data set was analysed with STATA 9.0 software (Stata Corp., College Station, TX). Descriptive statistics were used to compute malariometric indices and a survey chi-square test ("svytab" command in STATA) was used to test for significant differences (p < 0.05) in proportions. A survey logistic regression ("svylogit" command in STATA) was used to carry out a multivariate adjusted analysis for the risk of malaria infection taking into account the cluster effect.

An alternative analytical approach was also carried out by using the Classification and Regression Trees (CART) software to analyse risk factors and identify interactions. Tree-based models (such as CART) are non-linear and non-parametric alternatives to linear models for regression and classification problems. CART models are fitted by binary recursive partitioning of a multidimensional covariate space, in which the dataset is successively split into increasingly homogeneous subsets until a specified criterion is stratified [13-17]. The one-standard error rule was applied to select the best tree, i.e. the smallest tree within 1 standard error of the minimum error tree was selected.

For the survey, a wealth indicator was defined as a proxy for household economic status, and constructed by combining household information on assets (ownership of radio, television, motorbike, and brick house) by using principal components analysis, a method extensively described elsewhere [18,19]. The index was the first principal component which explained 42% of the variability among the four variables, and gave greatest weight to ownership of a TV (0.62), of a motorbike (0.60), then of a brick house (0.48); ownership of a radio had a much lower weight (0.16). The wealth index was then divided into 3-quantiles so that each household was divided into lower- medium- and higher wealth category, with median scores of -1.08, 0.29, and 1.82, respectively.

### Ethical considerations

The study was approved by the ethical committees of both the Institute of Tropical Medicine, Antwerp, Belgium and of NIMPE, Hanoi, Vietnam. Moreover, the Vietnamese Ministry of Health gave its permission to carry out the study. The fundamental principles of ethics in research on human participants were upheld throughout the project. The research procedures were disclosed to the participants and informed consent was sought from them or their legal representatives. Nobody was coerced into the study and if individuals wished to withdraw, they were allowed to do so without prejudice.

## Results

In March 2004, there were 18,646 people in the study area (Table 1). The main ethnic group, representing almost 90% of the population, was Ra-glai. The population was young, with more than half under the age of 20 years (median age: 19 years), and uneducated. Almost all households had forest fields (97.2%, 3,548/3,652), and more than half of the people were forest workers, mainly working in the forest fields (97.9%).

**Table 1:** Baseline characteristics of the study population

**Study population, N = 18,646**	**n**	**%**
**Sex (ratio = 0.98)**		
- Male	9,230	49.5
**Age groups:**		
- < 10 y	5,026	27.0
- 10–19 y	4,619	24.8
- 20–39 y	5,351	28.7
- 40–59 y	2,768	14.9
- > 59 y	882	4.7
**Ethnic groups:**		
- Ra-glai	16,438	88.2
- K'ho	1,728	9.3
- Kinh	454	2.4
- Others (Chu, Cham, Ede)	26	0.1
**Education level (age ≥ 20, n = 9,001):**		
- None	4,230	47.1
- Primary school	4,101	45.6
- Secondary school or higher	654	7.3
- Missing	16	0.2
**Occupation:**		
- None (children, students, retired people)	8,229	44.1
- Forest work (farming & other)	9,868	52.9
- Other (teacher, health staff...)	532	2.9
- Missing	17	0.1
**Bed net use in the village:**		
- Sleep under ITN	16,088	86.3
- Sleep under an untreated bed net	1,310	7.0
- Sleep without bed net	1,230	6.6
- Missing	18	0.1
**Forest activities:**		
- Never	7,864	42.2
- Only during day	6,425	34.5
- Work and sleep in the forest	4,340	23.3
- Missing	17	0.1
**Days/month spent in forest, **median [range] (n = 10,728)	24 [1;30]	
**Nights/month spent in forest, **median [range] (n = 4,340)	15 [1;30]	
**Bednet/hammock use in the forest (n = 4,340):**		
- Sleep under ITN	2,498	57.6
- Sleep in a hammock	549	12.6
- Sleep under ITN and hammock	635	14.6
- Sleep without bed-net and hammock	658	15.2

***Households N = 3,652 ***	*n*	*%*
**House structure:**		
- Thatched bamboo	1,594	43.7
- Wooden boards	1,006	27.6
- Dried mud	496	13.6
- Bricks	556	15.2
**Socio economic level:**		
- No radio, TV, motorbike	1,437	39.4
- Only a radio	1,160	31.8
- Only TV	204	5.6
- TV + radio (no moto)	268	7.3
- **At least a motorbike (+/-radio, TV)**	583	16.0

In total, more than a third (34.5%) of the population had only daily activities in the forest, while another 23% was working and sleeping there overnight, with a substantial number of days/nights spent in the forest (respective medians were 24 and 15 days).

ITN use in the villages was high (86.3%), and a few additional people were sleeping under an untreated bed net, with a median of 2.5 people per bed net, regardless of insecticide treatment. Hammocks were also popular: half of households had hammocks (50.1%) with a median of one hammock per household. Among people staying overnight in the forest, ITN use was lower (72.2%) than in those just sleeping in the villages, hammocks were used by 27.2% of the people (with or without ITN); 15.2% of the forest workers were sleeping without hammocks or ITNs.

Socio-economic status was generally low with 43.7% (1,594/3,652) houses made of thatched bamboo and only 15.2% (556/3,652) of bricks. Ownership of radios, TV and motorbikes was relatively low with 39.4% (1,437/3,652) of households having none of them (Table 1).

Three thousand twenty three individuals aged 10–60 years were included in the malariometric survey (out of 3,200 people randomly selected from the census file), a 94.5% participation rate (Table 2). The spleen rate was 1.2% (35/3023) and the parasite rate (all species) was 14.2% (429/3,026), with a high proportion of asymptomatic infections (87.9%). *Plasmodium falciparum *and *Plasmodium vivax *infections were equally represented, although the mean parasite density was significantly lower for the latter (Table 2).

**Table 2:** Malariometric indices

**Indicators (N = 3,023 participants)**	**n,**	**%**	**(95%CI)**
History of fever in 48 hours	266	8.8	(4.8 ; 2.9)
Fever present	72	2.4	(1.2 ; 3.5)
Spleen rate	35	1.2	(0.0 ; 2.5)
Malaria infection	429	14.2	(9.6 ; 18.8)
*P. falciparum*	*197/429*	*45.9*	*(41.8 ; 50.1)*
*P. vivax*	*186*	*43.4*	*(38.1 ; 48.7)*
*P. malariae*	*3*	*0.7*	*(0.0 ; 1.7)*
*Mixed infections*	*43*	*10.0*	*(6.9 ; 13.1)*
Asymptomatic infections	377	87.9	(81.3 ; 94.5)
Parasite density/(geometric mean):			
*P. falciparum*	139.2		(111.5 ; 173.7)
*P. vivax*	67.7		(57.8 ; 79.4)
*P. malariae*	140.7		(54.1 ; 365.5)

Uni- and multi-variate adjusted risk factor analysis showed that women, adults, educated, wealthy people and people sleeping under an ITN had significantly lower risk of having a malaria infection (Table 3). As expected, forest workers sleeping in the forest had a two- to three-fold significantly higher risk of malaria infection (adjusted-OR: 2.70; 95%CI [1.4; 5.2]). Ra-glai people tended to be more infected compared to the other ethnic groups, although significance was not reached. Housing conditions could not be included in the multivariate analysis since it was correlated with the wealth indicator. Moreover, multiple interactions occurred between wealth and bed net use, wealth and forest work, bed net and ethnicity, etc. The CART analysis included all risk factors identified by multivariate analysis, as well as house structure.

**Table 3:** Risk factor analysis for malaria infection: uni- and multivariate adjusted analysis using survey logistic regression (n = 429)

**Risk factors**	**Parasite prevalence**	**Cases**		**Unadjusted**	**Adjusted**
	
	**(%)**	**n**	**N**	**OR (95%CI)**	
**Total**	14.2	429	3,023		
**Gender:**					
- Male	16.0	225	1,409	1.00	1.00
- Female	12.6	204	1,614	0.76 (0.59–0.99)	0.75° (0.57–0.98)
**Age (y):**					
- < 16	17.9	121	677	1.00	1.00
- 16–45	13.7	270	1,976	0.73° (0.59–0.90)	0.65° (0.52–0.81)
- > 45	10.3	38	370	0.53° (0.36–0.76)	0.47° (0.31–0.73)
**Ethnic group:**					
- Ra-glai	15.1	409	2,708	1.00	1.00
- Others	6.3	20	315	0.39 (0.14–1.10)	0.47 (0.16–1.35)
**Education:**					
- No	16.4	165	1,007	1.00	1.00
- Primary	14.2	250	1,760	0.84 (0.55–1.31)	0.85 (0.55–1.33)
- Secondary or more	5.5	14	255	0.30° (0.20–0.44)	0.42° (0.27–0.64)
**Wealth:**					
- Low	17.1	305	1,783	1.00	1.00
- Medium	13.6	40	295	0.76 (0.30–1.92)	0.87 (0.36–2.10)
- High	8.9	84	945	0.43° (0.32–0.71)	0.51° (0.35–0.75)
**Forest activities last month:**					
- No work & no sleep in forest	12.5	50	401	1.00	1.00
- Work +/- sleep occasionally	14.1	358	2544	1.15 (0.67–1.96)	1.19 (0.73–1.92)
- Work & sleep regularly	26.9	21	78	2.59° (1.29–5.19)	2.70° (1.40–5.21)
**Bed net use:**					
- No	28.5	39	137	1.00	1.00
- Untreated bed net	23.3	49	210	0.76 (0.42–1.38)	0.82 (0.44–1.53)
- Insecticide-treated bed net	12.7	341	2,674	0.37° (0.17–0.78)	0.46° (0.23–0.90)

°p < 0.05

**Figure 1:**
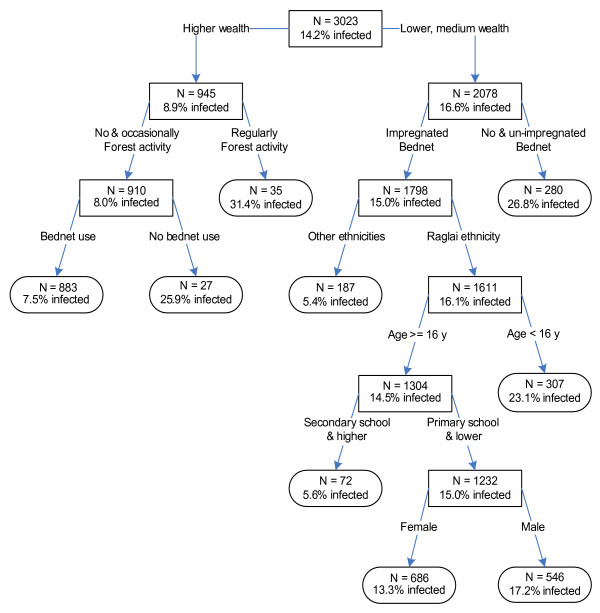
Classification tree of the risk factors for malaria infection.

According to the overall discriminatory power in the CART analysis, wealth emerged as the strongest overall discriminating risk factor for malaria infection, followed by educational level, bed net use, ethnicity, forest activity and house structure; age and gender were the last two factors (Table 4). The classification tree partitioned the different risk factors according to the overall discriminatory power of variables (Figure 1). Each class was then divided in two other sub-classes, either high (> 14%) or low malaria (< 14%) prevalence. Among wealthy people, where malaria prevalence was 9% (compared to 17% in low-medium wealth), the next most important risk factor was regular forest activity (malaria prevalence: 31.4%) while for those without regular forest activity, bed net use reduced significantly the risk (malaria prevalence: 7.5% *versus *26% in non-users) (Figure 1). In people of low-medium wealth, ITN use was the most important risk factor and reduced significantly the malaria risk. Among those using ITN, belonging to the Ra-glai ethnic group and being less than 16 years of age were important risk factors for malaria infection, while for adults Ra-glai malaria prevalence was higher in those with a lower education (15% *versus *5.6% in higher education), with a slightly disadvantage for men (17% *versus *13%, Figure 1). House structure did not appear as a main splitter in the tree but was an important surrogate splitter as shown by its overall discriminatory power of almost 50% (Table 4).

**Table 4:** Ranking of malaria risk factors by overall discriminatory power

***Variable***	***Power***
Wealth	100.0
Education	83.7
Bednet use	75.8
Ethnicity	56.9
Forest activity	55.8
House structure	48.9
Age	32.2
Gender	10.2

In a subsequent multivariate analysis, stratifying by wealth group, house structure was indeed identified as the strongest risk factor in the low/medium wealth group beside all other risk factors reported by CART, brick houses being much less associated with malaria than bamboo ones (Adjusted OR: 0.26; 95% CI [0.09; 0.80], p = 0.021). As shown by CART, in this socio-economic group, forest activity was not a risk factor since malaria prevalence was not significantly different among people never working in the forest and those working and sleeping there regularly (16.5% versus 13.3%, p = 0.68). In contrast, forest activity was a very strong risk factor for malaria in the wealthiest group, (adjusted OR: 7.71; 95%CI [3.46; 17.16], p < 0.001), and bed net use (ITN and non-treated nets) was highly protective (adjusted OR: 0.22; 95%CI [0.09; 0.53], p = 0.02). A strong interaction occurred between bed net use and forest activity. However, due to a lack of events in some categories, it could not be handled by the model. Interestingly, house structure was not associated with an increased risk of malaria in the wealthiest group.

## Discussion

Malaria is still endemic in the forested mountainous areas of Ninh Thuan province, where ethnic minorities, such as the Ra-glais, usually have a low economic status, low education and are frequently exposed to malaria because of their forest activities [7,11]. The relatively high transmission is confirmed by the high number of asymptomatic infections detected during the cross-sectional survey. This parasite reservoir contributes maintaining malaria transmission and does represent a threat for other provinces where the malaria risk is almost zero. The risk factors for malaria infection, i.e. age < 16 years, belonging to an ethnic minority, in this case Ra-glai, low education, poverty, forest activity and no bed net use, are similar to those identified in this area and reported by previous studies [10,11], both by uni- and multi-variate analysis. Regular forest activity is confirmed to be the strongest risk factor for malaria infection, an almost three-fold higher risk by multivariate analysis in people regularly working and sleeping in the forest compared to those not going to the forest. However, the multivariate analysis was limited since it could not handle the multiple interactions that occurred in the model, especially between wealth and forest activity, wealth and bed net use, bed net use and ethnic group, etc... Therefore, the effect of the different risk factors identified by survey logistic regression was not similar across categories of the other risk factors, i.e. the effect of forest activity or bed net use was different for different wealth categories or ethnic groups, but this could not be shown in the multivariate analysis.

Risk factor analysis handling multiple interactions can be carried out by using a based-tree model such as the CART that expresses its result in the form of a decision tree, a completely different approach than the usual statistical analysis. Indeed, in the classical regression the linear combinations are the primary method of expressing the relationships between variables while in CART this does not need to be linear or additive and the possible interactions do not need to be pre-specified or of a particular multiplicative form. Therefore, the classification tree provides a much more flexible relationship between variables; missing values of the covariates, multi-colinearity and outliers are taken care of in an intuitively correct way [13]. Outliers for example are isolated into a node and thus have no effect on splitting. Missing values in predictor variables can be estimated from other predictor ("surrogate") variables so that partial data can be used whenever possible within the tree. The overall discriminatory power of each explanatory variable can also be determined. The tree allows then to explore the relationship between different risk factors and their relative importance, something that it is not possible with the classical multivariate analysis. Wealth appeared to be the most important risk factors for malaria infection with individuals of low-medium wealth being more at risk. Surprisingly, in this group forest activity did not appear to be the most important risk factor while in the wealthier group, regularly working and sleeping in the forest dramatically increased the risk of malaria. In the low-medium wealth group, the malaria risk was already high regardless of forest activity, possibly because of poorer housing conditions increasing their exposure to infective bites within their villages. House structure was identified as an important risk factor for malaria as shown by the ranking of its discriminatory power, despite the fact that it did not appear as a main splitter in the final tree. This happens because house structure is an important "surrogate" but not a major splitter. Indeed, the ranking by overall discriminatory power is determined by the sum across all nodes in the tree of the improvement scores that the predictor has when it acts as a primary or a surrogate splitter. Thus, house structure enters the tree as the top surrogate splitter in many nodes but never as a primary splitter. In the subsequent multivariate analysis stratified by wealth, house structure was indeed a strong risk factor in the low-medium wealth but not for the wealthier group, where regularly sleeping in the forest and bed net use were most important.

Therefore, CART can give further insights on results produced by multivariate analysis and reciprocally multivariate analysis can quantify CART results leading to a more refined understanding of the actual importance and interplay between risk factors.

Indeed, in the initial multivariate analysis, adjustment for housing condition was not possible due to co-linearity with the wealth indicator. However, after the CART analysis, the hypothesis of housing conditions as a strong malaria risk factor could be checked and quantified with a stratified multivariate analysis in the group of low-medium wealth. Results suggest that improved housing conditions might achieve a protective effect against malaria in poor rural areas of Central Vietnam, as it has been described elsewhere for other countries [20,21]. Thus, the current poverty alleviation program launched by the Vietnamese government [22] and consisting, among others, in providing brick houses for the poorest, might have a positive impact on malaria prevalence.

As shown by the CART and the multivariate analysis, bed net use was significantly protective for both income levels groups. Nevertheless, bed nets in the wealthiest group, regardless of insecticide treatment, were highly protective except for people regularly sleeping in the forest, while in the low-medium wealth group only ITN were protective. Overall, the effect of ITNs in the low-medium wealth group seems to be weaker than that of any bed net in the wealthier group.

Among ITNs users, Ra-glais were much more infected than other ethnic groups, especially those less than 16 years of age. Probably, this could be explained by the Ra-glai way of life which is deeply interwoven with the forest life (impossible to detail in a large cross-sectional survey) since very early in life (babies carried on their mother's back). Therefore, in Ra-glais the risk of malaria infection is much higher and at an earlier date than in other ethnic groups. The strong difference in malaria prevalence between children and adults indicates the development of protective immunity with age, a consequence of the early and relatively intense exposure to infection. Hopefully, more detailed information on this aspect will be generated by results of the 2-year serological follow-up of the study cohort.

In conclusion, the CART approach is useful and complementary to the classical multivariate analysis. Indeed, CART can handle multi-colinearity, multi-level interactions, missing values and can identify malaria risk factors potentially vulnerable to control activities with their expected impact. This is not possible with the multinomial models (e.g. logistic regression) as they do not rank risk factors according to their importance, particularly when multiple interactions or co-linearity occur. Combining both techniques allows for a much more refined analysis and new insights on the main determinants of malaria infection. Results of this study show that malaria control in these areas remains an extremely complex task, not only limited to protect people sleeping in the forest, but the population as a whole, whose vulnerability to malaria greatly depends on poverty-related risk factors such as education, ethnicity or housing conditions.

## Authors' contributions

TND contributed to the study design, study coordination and supervision, field work, data entry, cleaning and analysis, and paper writing; AE contributed to the study design, study coordination and supervision, field work, statistical analysis and reviewed the manuscript; NS contributed to the data analysis and reviewed the manuscript; HLX contributed to the study design, study coordination and supervision, and reviewed the manuscript; TLK contributed to the study design and reviewed the manuscript; HTC contributed to the field work; KPV contributed to the study coordination and supervision, field work, data entry and cleaning; MC reviewed the paper; UDA contributed to the study design, study coordination and supervision, data analysis and reviewed the manuscript; All authors read and agreed with the final manuscript
